# Genome-Wide Identification and Expression Analysis of the *HSF* Gene Family in *Ammopiptanthus mongolicus*

**DOI:** 10.3390/cimb46100678

**Published:** 2024-10-14

**Authors:** Shuai Zhao, Jun Qing, Zhiguo Yang, Tian Tian, Yanqiu Yan, Hui Li, Yu’e Bai

**Affiliations:** 1 College of Forestry, Inner Mongolia Agricultural University, Hohhot 010019, China; zhaoshuaifight@163.com (S.Z.);; 2 Institute of Desertification Studies, Inner Mongolia Academy of Forestry, Hohhot 010019, China

**Keywords:** *Ammopiptanthus mongolicus*, *HSF* gene family, heat stress, expression pattern

## Abstract

*Ammopiptanthus mongolicus* is an ancient remnant species from the Mediterranean displaying characteristics such as high-temperature tolerance, drought resistance, cold resistance, and adaptability to impoverished soil. In the case of high-temperature tolerance, heat shock transcription factors (HSFs) are integral transcriptional regulatory proteins exerting a critical role in cellular processes. Despite extensive research on the HSF family across various species, there has been no analysis specifically focused on *A. mongolicus*. In this study, we identified 24 members of the *AmHSF* gene family based on the genome database of *A. mongolicus*, which were unevenly distributed over 9 chromosomes. Phylogenetic analysis showed that these 24 members can be categorized into 5 primary classes consisting of a total of 13 subgroups. Analysis of the physical and chemical properties revealed significant diversity among these proteins. With the exception of the AmHSFB3 protein, which is localized in the cytoplasm, all other AmHSF proteins were found to be situated in the nucleus. Comparison of amino acid sequences revealed that all AmHSF proteins contain a conserved DNA-binding domains structure, and the DNA-binding domains and oligomerization domains of the *AmHSF* gene exhibit conservation with counterparts across diverse species; we investigated the collinearity of *AmHSF* genes in relation to those of three other representative species. Through GO enrichment analysis, evidence emerged that *AmHSF* genes are involved in heat stress responses and may be involved in multiple transcriptional regulatory pathways that coordinate plant growth and stress responses. Finally, through a comprehensive analysis using transcriptome data, we examined the expression levels of 24 *AmHSFs* under 45 °C. The results revealed significant differences in the expression profiles of *AmHSFs* at different time intervals during exposure to high temperatures, highlighting their crucial role in responding to heat stress. In summary, these results provide a better understanding of the role and regulatory mechanisms of HSF in the heat stress response of *A. mongolicus*, meanwhile also establishing a foundation for further exploration of the biological functions of *AmHSF* in the adversity response of *A. mongolicus.*

## 1. Introduction

Plants reside within a dynamic environment, enduring numerous stresses throughout their growth and development. These stresses comprise both biotic factors, such as pathogen infections and herbivore attacks, and abiotic stresses, including heat, drought, cold, and nutrient deficiencies [1]. “Heat stress” encompasses heat shock, heat waves, and warming experiments, each exhibiting variations in the duration and intensity of temperature elevation. These factors can influence plants at any stage of their developmental processes [2]. In numerous molecular investigations, “heat stress” is interchangeable with “heat shock”, which refers to the short-term exposure of a plant to severe high temperatures [3]. Such stress typically persists for several minutes to a few hours, with air temperature increases frequently exceeding 20 °C above the optimal range necessary for the plant’s growth or development [4]. Following heat stress, plants enhance their thermotolerance by regulating heat shock proteins and modulating the secretion of plant hormones such as gibberellin (GA), jasmonic acid (JA), abscisic acid (ABA), and indole-3-acetic acid (IAA) [4]. These processes are closely associated with heat shock transcription factors.

The heat shock factor (HSF) transcription factor family is one of the extensively studied transcription factors in plants, with a widespread distribution in both eukaryotes and prokaryotes [5]. The initial discovery of an *HSF* gene occurred in the yeast *Saccharomyces cerevisiae* in 1988 [6], after which *HSF* genes were subsequently cloned in fruit flies and mammals. The pioneering cloning of *HSF* genes in plants was first achieved in tomatoes [7], subsequently expanding to model plants including *A. thaliana* [8] and rice [9]. As plant genomic information is continually deciphered and refined, *HSF* gene family’s identification and analysis have expanded to encompass over 30 plant species. This comprehensive scope encompasses common plants such as Arabidopsis [10], rice [11], maize [12], *Populus trichocarpa*, *Medicago truncatula* [13], cucumber [14], and *Glycine max* [3]. The quantity and variety of HSF family members varies significantly among plant species, with wheat (*Triticum aestivum*) boasting the highest recorded count of 56 members [15,16], while Alfalfa possesses a minimum of 16 members. Terminal components in the signal transduction chain for stress-responsive gene expression, HSF transcription factors function in mediating the activation of genes in response to heat or other stress conditions [12], such as heat shock proteins (HSPs), ascorbate peroxidase (APX), and catalase (CAT) [2,3]. Research has revealed that *HSFs* are involved in various stress responses, including high-temperature stress, salt stress, drought stress, and oxidative stress [17,18,19].

*HSFs* constitute a highly conserved and extensive gene family characterized by a complex structure in plants compared to other eukaryotes [20,21]. The primary structure of HSF in plants comprises five prominent domains: DNA-binding domain (DBD), oligomerization domain (OD or HR-A/B region), nuclear localization signal (NLS), nuclear export signal (NES), and C-transcriptional activation domain (CTAD) [20,21]. The remarkably conserved DBD is positioned near the N-terminus of the identified HSFs and consists of a three-helix bundle and a compact four-stranded antiparallel β-sheet, forming an aquaphobic core. This central and conserved region features a helix-turn-helix (HTH) motif variant structure analogous to that of the leucine zipper family of transcription factors, which enables precise binding to heat shock element (HSE) sequences (5′-AGAANNTTCT-3′). The DBD plays a vital role in activating or inhibiting the transcription of stress-related genes [22,23]. Adjacent to the DBD, the OD contains two hydrophobic heptad-repeat regions: HR-A and HR-B. HR-A/B exhibits a coiled-coil structure formed by three helices and an α-helix structural region containing numerous hydrophobic amino acid residues. HR-A/B can connect to the DBD through a variable-length flexible linker (15–80 amino acid residues), facilitating interactions among proteins during transcriptional activation and engaging in import and export functions in nuclear [24,25]. Founded on the distinctive features of the OD, the HSF gene family is classified into three subfamilies, designated as A, B, and C. Subfamily A and C members possess insertions of 21 and 7 amino acids, respectively, between HR-A and HR-B, while subfamily B lacks insertion sequences between these two regions [24]. The nuclear localization signal (NLS) domain of HSFs is an essential structure for facilitating protein entry into the nucleus. It is adjacent to the C-terminus of HR-A/B and consists of a cluster of basic amino acid residues, particularly enriched in arginine and lysine. On the other hand, a NES is situated at the C-terminus of HSFs and contains an abundance of leucine residues, aiding in the export of HSFs from the nucleus to the cytoplasm. Most plant HSFs possess both nuclear localization signals and nuclear export signals. These structural domains regulate the balance of nuclear export and import during plant heat stress responses. Through their coordinated action, the distribution of HSFs within the cell is determined [20]. The CTAD is the least conserved region in the HSF sequence, containing a short peptide AHA motif (aromatics, hydrophobic, and acidic amino acid residues) and abundant aromatic, hydrophobic, and acidic amino acid residues. The AHA motif is a unique domain specific to the HSFA subfamily; its presence varies among different HSFAs. This motif plays a decisive role in determining the transcriptional activation of HSFA. Conversely, the absence of the AHA motif in the HSFB and HSFC subfamilies indicates their lack of involvement in transcriptional activation activity [20,26]. Notably, the HSFB subfamily (excluding HSFB5) contains a specific four-peptide motif, LFGV, with inhibitory effects [27]. In summary, DBD is the most conserved region in the HSF protein structure. OD and AHA domains are variable, reflecting functional differences among the three HSF subfamilies [27]. Generally, A-class HSFs are considered the primary regulators in heat stress, inducing the expression of stress-responsive genes. In contrast, B-class HSFs, under normal conditions, inhibit the transcription expression of heat-tolerant genes and play a crucial role in plant heat tolerance defense. Research on C-class HSFs is relatively limited, but they are also involved in stress responses [28].

*Ammopiptanthus mongolicus* is an evergreen broad-leaved shrub belonging to the legume genus *Ammopiptanthus* Cheng f. It was discovered in the Central Asian desert. *A. mongolicus* represents a remnant species from the third generation of ancient Mediterranean coastal plants, surviving the retreat of the ancient Mediterranean and the subsequent climate aridification process. *A. mongolicus* is the only evergreen broad-leaved plant distributed in the Central Asia desert and plays a crucial role in windbreak and sand-fixing vegetation in harsh and barren desert areas. Even during winter and spring when strong winds reduce litter coverage from other shrubs, it remains a valuable protective asset [29]. *A. mongolicus* possesses desirable traits, such as high-temperature tolerance, drought resistance, cold resistance, and adaptability to nutrient-poor soils, making it a crucial subject for studying plant adaptability and stress resistance mechanisms [30]. Despite increasing interest in studying the stress-responsive genes and molecular mechanisms of *A. mongolicus* in adapting to challenging environments, there is limited research on its heat tolerance mechanisms and the *HSF* gene family. In this study, we employed conserved motifs, gene structures, and phylogenetic relationships to characterize the *AmHSF* gene family using RNA-seq data from *A. mongolicus*. The expression patterns of *AmHSF* were analyzed under high-temperature stress at different time points. These findings enhance our comprehension of the evolutionary connections and functional distinctions within the *AmHSF* gene. Furthermore, they provide a foundation for additional investigation into the functions of *AmHSF* and the exploration of their regulatory mechanisms in *A. mongolicus*.

## 2. Materials and Methods

### 2.1. Identification and Sequence Characterization of AmHSF Transcription Factors

To identify *HSF* genes in *A. mongolicus*, the HSF protein DNA-binding domain (DBD) structure domain (PF00447) HMM profile was downloaded from the Pfam website (https://www.ebi.ac.uk/interpro/entry/pfam/#table, accessed on 9 April 2024). Employing HMMER3.1 software and applying a threshold e-value of <1 × 10^−5^, we detected candidate gene family members harboring the HSF domain. Meanwhile, the HSF protein sequences of *Arabidopsis thaliana* sourced from the TAIR database (https://www.arabidopsis.org/) served as reference sequences. Subsequently, the protein sequences encoded by *A. mongolicus* genomic data obtained from Rthe National Genomics Data Center (https://ngdc.cncb.ac.cn/gwh/Assembly/84098/show, accessed on 9 April 2024) were subjected to BLASTp analysis against the protein sequences of *AtHSF* by a threshold e-value < 1 × 10^−5^. Finally, we combined the results of BLASTp and Hmmer searches. We further validated and identified members of the *A. mongolicus* HSF family using WebCD-search (https://www.ncbi.nlm.nih.gov/cdd, accessed on 9 April 2024) and SMART (http://smart.embl.de/, accessed on 9 April 2024). After eliminating low-reliability and redundant sequences, a total of 24 *AmHSFs* were ultimately identified.

The physical and chemical properties of AmHSF proteins, including amino acid composition, molecular weight, and isoelectric point, were assessed using ProtParam (http://web.expasy.org/protparam/, accessed on 10 April 2024). The hydrophobicity, instability index, and solubility coefficient were inferred using the method described in [31] through ExPASy (https://web.expasy.org/protscale/, accessed on 10 April 2024). Subcellular localization predictions were conducted using PSORT (https://wolfpsort.hgc.jp/, accessed on 10 April 2024).

### 2.2. Alignment of Multiple Amino Acid Sequence and Phylogenetic Tree Construction of HSFs from Different Species

Multiple amino acid sequence alignment was conducted using ClustalW (https://www.genome.jp/tools-bin/clustalw, accessed on 12 April 2024). The conserved domains of AmHSF proteins, including the DNA-binding domain (DBD) and the HR-A/B region (OD), were aligned and visualized usingSnapGene 6.0.2 software (accession date: 12 April 2024).

To improve the exploration of the classification and phylogenetic relationships of AmHSF proteins, a total of 83 HSF protein sequences—including 21 *A. thaliana* HSF protein sequences, 38 *G. max* HSF protein sequences (https://www.soybase.org/, accessed on 12 April 2024), and 24 identified AmHSF protein sequences—were subjected to phylogenetic tree construction utilizing the neighbor-joining method in MEGA-11 software (accession date: 12 April 2024), where evolutionary distances were determined by the Poisson correction method with 1000 bootstrap replicates. Based on the zinc finger structure, AmHSF proteins were categorized into multiple subgroups. The final phylogenetic tree was further refined using iTOL (https://itol.embl.de/, accessed on 12 April 2024). IDs for AtHSF and GmHSF proteins can be found in Supplementary Table S1.

### 2.3. Gene Structure and Conserved Motif Analysis

The conserved HSF proteins sequences underwent analysis using the MEME online tool (https://meme-suite.org/meme/, accessed on 12 April 2024) with the following specified parameters: minimum width ≥ 6, maximum width 50, and 10 motifs. Subsequently, the GFF annotation file was utilized to obtain the intron–exon distribution of *AmHSF*. The visualization of gene structures and conserved motifs of *AmHSF* was conducted using TBtools software v2.019 [32].

### 2.4. Chromosomal Localization and Covariance Analysis

Chromosome lengths and gene locations were extracted from the genome annotation files of *A. mongolicu*. Gene positions on chromosomes were visualized using the Gene Location Visualize from the GTF/GFF plugin in TBtools (v2.019). MCScanX, with default parameters, was employed to identify gene duplication events of HSF genes within the *A. mongolicus* genome, as well as across the genomes of *A. mongolicus* and *A. thaliana*, *A. mongolicus* and *G. max*, and *A. mongolicus* and *Oryza sativa*. Circos plots were generated using the Advanced Circos plugin, and collinearity among different species was visualized with the Multiple Synteny Plot plugin in TBtools.

### 2.5. Identification of Cis-Acting Elements on Promoter of HSF

The 2000 bp region upstream of the HSF coding sequences was retrieved to perform promoter analyses using PlantCARE (https://bioinformatics.psb.ugent.be/webtools/plantcare/html/, accessed on 20 April 2024) to predict *Cis*-acting elements within the promoter regions essential for gene expression, development, and hormone signal transduction. The predicted *Cis*-acting elements were subsequently visualized utilizing the Simple BioSequence Viewer plugin of TBtools software.

### 2.6. AmHSF Expressing Pattern under High-Temperature Stress

Mature seeds of *A. mongolicus* used in this study were obtained from Ordos City, Inner Mongolia, China. Seedlings that had grown evenly for four months were selected for high-temperature stress treatment. The seedlings were randomly divided into six groups, with one group serving as the control. The remaining five groups were exposed to a high temperature of 45 °C for 3, 6, 12, 24, and 48 h, respectively, following normal watering the day before treatment. Sampling was conducted at the corresponding time points. Leaf samples from both control and stressed seedlings were collected, quickly frozen in liquid nitrogen, and stored at −80 °C. RNA extraction, quality assessment, library construction, and sequencing were performed by Metware Biotechnology Co., Ltd. (Wuhan, China). Gene expression levels were quantified using fragment per kilobase per million mapped reads (FPKM) values. Finally, the heat map was generated using the Heatmap program in TBtools.

### 2.7. GO Enrichment Analysis

To elucidate the potential biological functions of the *AmHSFs* and the biological processes in which they are involved, an online network (https://cloud.metware.cn/#/tools/detail?id=145, accessed on 23 April 2024) was utilized to perform gene ontology (GO) functional enrichment analysis.

### 2.8. qRT-PCR Analysis of AmHSF

In order to validate the accuracy of the RNA-Seq of *A. mongolicus* seedlings under high-temperature stress, 12 *AmHSF* genes were randomly selected for quantitative real-time PCR (qRT-PCR) analysis. Total RNA was extracted from *A. mongolicus* leaves using the OminiPlant RNA Kit (DNase I) (CW2598, CWBIO, Taizhou, China). Reverse transcription of 1000 ng of RNA was performed using the FastKing gDNA Dispelling RT SuperMix kit (KR220511, Tiangen, Beijing, China). Subsequently, real-time quantitative PCR (qRT-PCR) was performed using TB Green^®^ Premix Ex Taq™ II (Takara, Dalian, China). Primers for *HSF* genes were designed using Primer3Plus (https://www.primer3plus.com/), and the primer sequences used in this study are listed in Table S2. The qRT-PCR reaction system followed the procedures of the LightCycler480 (F. Hoffmann-La Roche AG, Basel, Switzerland) with the following conditions: 95 °C for 5 min; 45 cycles of 95 °C for 10 s, 60 °C for 15 s, and 72 °C for 15 s. The final extension step was at 95 °C for 10 s, 60 °C for 1 min, and 40 °C for 10 s. Each experiment was conducted with three replicates, and the gene relative expression was computed using the 2^−ΔΔCt^ algorithm. To validate the RNA-seq data, a correlation analysis was performed between the log_2_ (qRT-PCR) data from qRT-PCR and the log_2_ (FoldChange) data from RNA-seq.

## 3. Results

### 3.1. Identification and Phylogenetic Analysis of AmHSF

In this study, we used HMMER with an E-value threshold of <1 × 10^−5^ to identify HSF members in *A. mongolicus*. After removing redundant transcripts and retaining only the longest transcript, we identified 24 HSF gene members from an initial 37 candidates. These 24 *A. mongolicus* HSF genes were blasted to 21 *A. thaliana* HSF proteins (AtHSFs) to name them. Additionally, we employed SMART and WebCD-search to analyze conserved structural domains, confirming that all 24 *A. mongolicus* HSFs contain the HSF DNA-binding domain, thereby validating their classification as HSF members.

**Figure 1:**
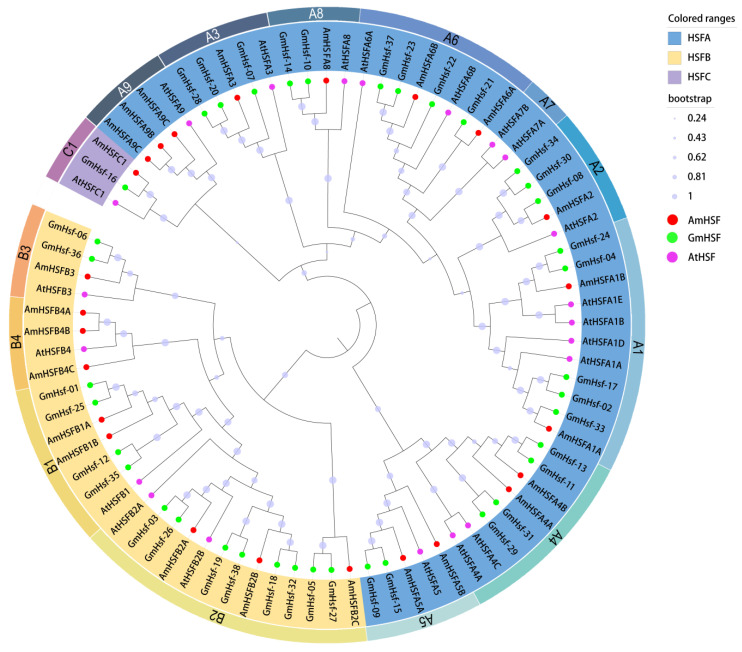
Phylogenetic tree of HSF proteins among *A. mongolicus*, *A. thaliana*, and *G. max*: In this graph, AmHSF proteins are denoted by red dots, AtHSF proteins are denoted by purple dots, and GmHSF proteins are denoted by green dots. Each protein class is distinguished by specific background colors: HSFA (blue), HSFB (yellow), and HSFC (purple).

To further validate the accuracy of the identified AmHSF family member and elucidate the evolutionary relationships among different species, a total of 83 HSF amino acid sequences, including 24 AmHSF proteins, 21 AtHSF proteins, and 38 GmHSF proteins, were subjected to multiple sequence alignment using ClustalW. Subsequently, the neighbor-joining (NJ) phylogenetic tree was constructed utilizing MEGA-11 software. The 24 proteins were categorized into 3 principal groups: Group A (green), Group B (yellow), and Group C (blue), encompassing 13 minor subgroups (A1, A2, A3, A4, A5, A6, A8, A9, B1-B4, and C1). Then, we named them as AmHSFA1A to AmHSFC1, based on protein structure and phylogenetic evolutionary tree branches. Among these, AmHSFAs were the largest subgroup with 14 members, namely AmHSFA1A, AmHSFA1B, AmHSFA2, AmHSFA3, AmHSFA4A, AmHSFA4B, AmHSFA5A, AmHSFA5B, AmHSFA6A, AmHSFA6B, AmHSFA8, AmHSFA9A, AmHSFA9B, and AmHSFA9C. AmHSF proteins were not aggregated into the A7 subgroups. AmHSFBs were divided into four subgroups (B1–B4) with nine members: AmHSFB1A, AmHSFB1B, AmHSFB2A, AmHSFB2B, AmHSFB2C, AmHSFB3, AmHSFB4A, AmHSFB4B, and AmHSFB4C. The smallest group was AmHSFC, which consisted of a solitary member, AmHSFC1. Following analysis of the phylogenetic tree results, it is evident that AmHSF proteins exhibit a closer clustering with GmHSF proteins, indicating notable evolutionary distinctions in HSF proteins between plants within the same and different families (Figure 1).

### 3.2. Physicochemical Analysis of AmHSFs

The anticipated physicochemical properties indicated that the 24 HSF proteins spanned from 132 to 561 amino acids (aa) in length. Among them, AmHSFB3 is the smallest (132 aa), while AmHSFB1A is the largest (561 aa). The relative molecular weight (MW) of these proteins varies from 15.11 kDa (AmHSFB3) to 63.11 kDa (AmHSFB1A), with an average MW of 41.79 kDa. The forecasted isoelectric point (pI) varies from 4.86 (AmHSFA8) to 8.87 (AmHSFB4A), with an average pI of 5.96. The instability index suggested that 21 out of 24 HSF proteins are unstable, except for the proteins encoded by AmHSFA5B, AmHSFB1A, and AmHSFB1B, which have instability indices below 40, making them stable proteins. Additionally, the aliphatic index (A.I.) ranges from 55.38 (AmHSFA5B) to 92.20 (AmHSFB3), suggesting minimal variations in their thermal stability. The average hydrophilic (GRAVY) scores were negative, suggesting that they are predominantly hydrophilic. Predictions of subcellular localization displayed that all HSF proteins, except for AmHSFB3, are projected to locate in the cell nucleus (Table 1).

### 3.3. Protein Characteristics of A. mongolicus HSF Family

**Figure 2:**
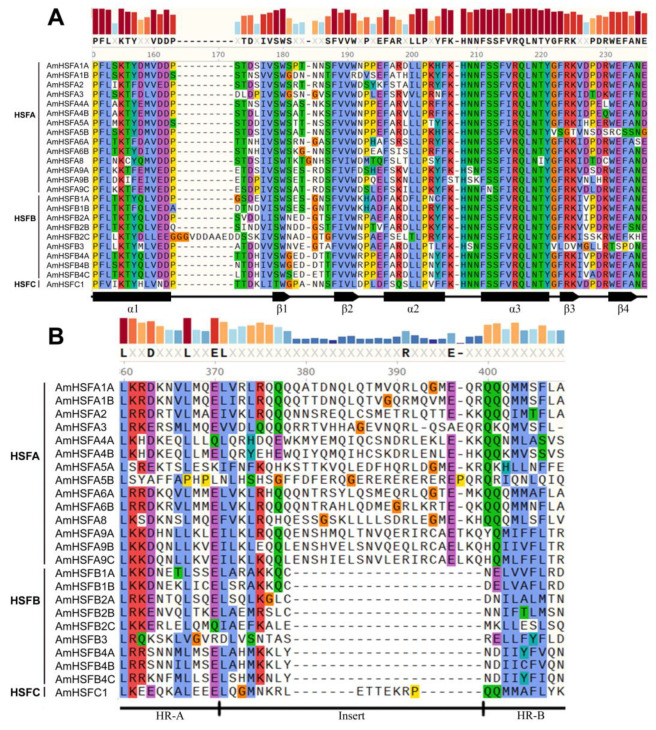
Visualization of AmHSF protein DNA-binding domain and oligomerization domain: (**A**) DNA-binding domain; (**B**) oligomerization domain. In this graph, the same color block denotes conserved amino acid sequence.

To further resolve the AmHSF protein class, the conserved DBD domain sequences and oligomerization domains for each member were extracted and visualized (Figure 2). In agreement with previous studies, the HSF domain of the AmHSF family members comprised three α-helical bundles (α1, α2, and α3) and four antiparallel β-sheets (Figure 2A), which are essential for the recognition and interaction of HSF proteins. Additionally, distinct characteristic features of the OD were observed across various subgroups of the AmHSF family (Figure 2B). While the OD of B-group HSF proteins display a compact structure in the HR-A/B region without any amino acid insertions, A-group and C-group proteins feature oligomerization domains with insertions of 21 and 7 amino acid residues, respectively (Figure 2B).

### 3.4. Gene Structure and Conservation Motif Analysis

**Figure 3:**
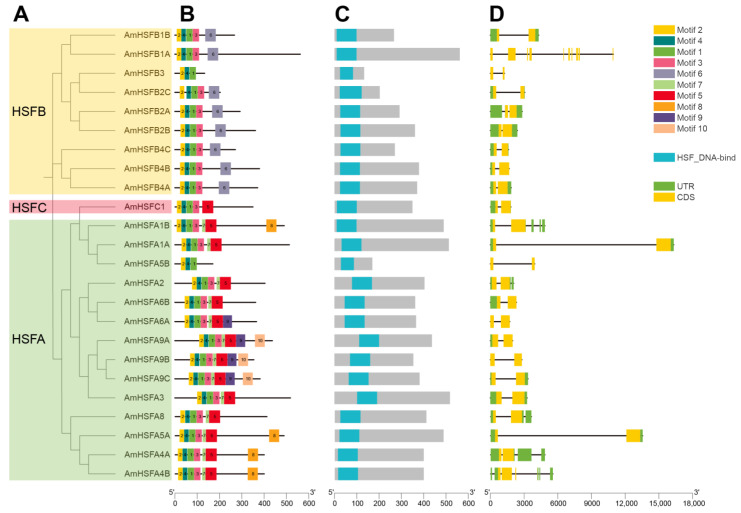
Gene structure and conserved motifs analysis of *AmHSFs*: (**A**) phylogenetic relationship tree; (**B**) conserved motifs of *AmHSFs*; (**C**) SMART conserved HSF domain; (**D**) gene structure of *AmHSFs*.

Based on the aforementioned phylogenetic analysis, we categorized *AmHSF* genes into three classes: HSFA, HSFB, and HSFC (Figure 3A). Using MEME analysis, ten highly conserved motifs were identified within each AmHSF protein (Figure 3B). The results indicated that the positions of the majority of motifs were consistent within each respective group. Utilizing the SMART and Pfam databases for a comprehensive search and analysis, we annotated Motif 1, Motif 2, and Motif 4 as segments of the HSF domain, while Motif 3 was annotated as HALZ. The remaining motifs lacked specific annotations. As anticipated, the presence of Motifs 1, 2, and 4 in all AmHSF proteins corresponds to the formation of a highly conserved HSF DBD region, indicating their potential importance in the biological functions of HSF proteins. (Figure 3C). Furthermore, Motif 5 was found exclusively in Class A and Class C proteins; Motif 6 was present only in Class B proteins; Motif 7 appeared solely in Class A proteins; Motif 8 was identified in the A1, A4, and A5 subgroups; Motif 9 was primarily distributed in the A6 and A9 subgroups; and Motif 10 was unique to the A9 subgroup proteins. These findings suggest that AmHSF proteins may share similar biological functions, while Motifs 5–10 could be linked to specific biological roles across different protein subgroups.

The gene structure of *AmHSF* genes revealed that *HSF* genes within the same class typically exhibit a comparable number of introns in their architecture. The outcomes of the gene structure analysis revealed that *AmHSF* genes typically contain at least two exons and one intron (Figure 3D). Specifically, the number of introns in the HSFA family genes ranges from one to five; a total of nine genes (*AmHSFA1A*, *AmHSFA3*, *AmHSFA5A*, *AmHSFA5B*, *AmHSFA6A*, *AmHSFA6B*, *AmHSFA9A*, *AmHSFA9B*, and *AmHSFA9C*) have one intron, and two genes (*AmHSFA2* and *AmHSFA8*) have two introns. One gene (*AmHSFA4A*) has three introns, one gene (*AmHSFA1B*) has four introns, and one gene (*AmHSFA4B*) has five introns. In the HSFB family genes, except for *AmHSFB1A*, which has the most (10) introns, and *AmHSFB2A*, which has two introns, the rest of the genes are two exons and one intron, and the HSFC family also has two exons and one intron.

### 3.5. Chromosomal Localization and Collinearity Analysis of AmHSFs in A. mongolicus

**Figure 4:**
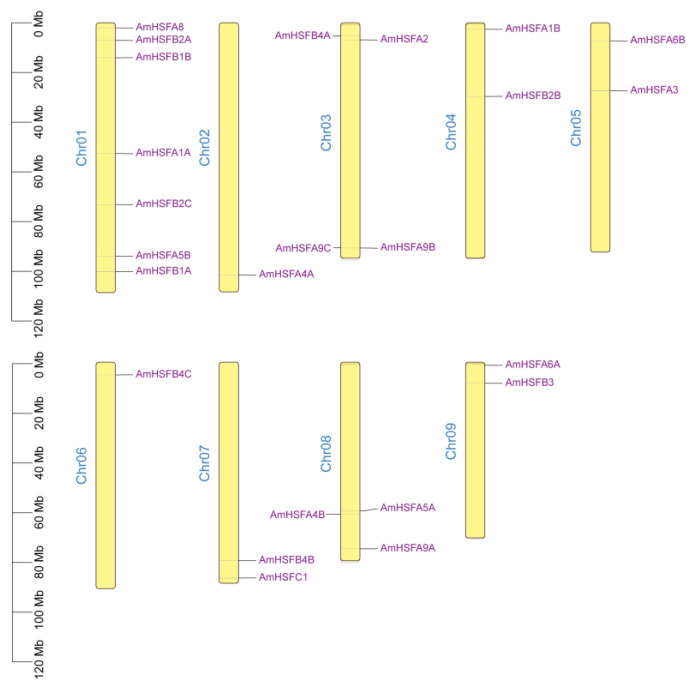
Chromosomal distribution of *AmHSFs* members, where yellow lines represent nine chromosomes and distribution of genes on the upper and lower arm.

**Figure 5:**
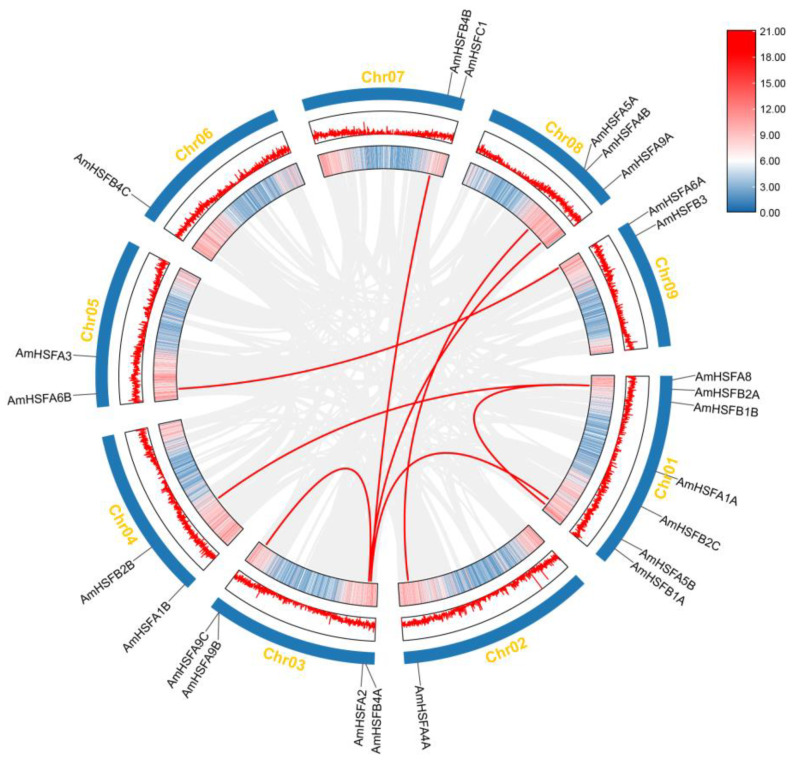
Chromosome distribution and interchromosomal relationships of *AmHSF* genes; the gray lines represent syntenic blocks within the *A. mongolicus* genome, while the red lines denote pairs of duplicated *AmHSF* genes.

Seven *HSFs* were located on chromosome I, representing approximately 29.14% of the total *HSF* genes. Chromosomes III and VIII contain four and three *HSFs*, respectively, while chromosomes IV, V, VII, and IX each harbor two *HSFs*. Chromosomes II and VI each have one *HSF* gene (Figure 4). Gene duplication events are prevalent across all species, as they can give rise to new functional genes and facilitate evolutionary processes. Consequently, we employed MCScanX genomic homology analysis to investigate tandem duplications within the *HSF* gene family of *A. mongolicus*. Two tandem duplicated genes (AmHSFA9B and AmHSFA9C) and eight collinear gene pairs of *A. mongolicus* involving eight chromosomes were identified (Figure 5).

**Figure 6:**
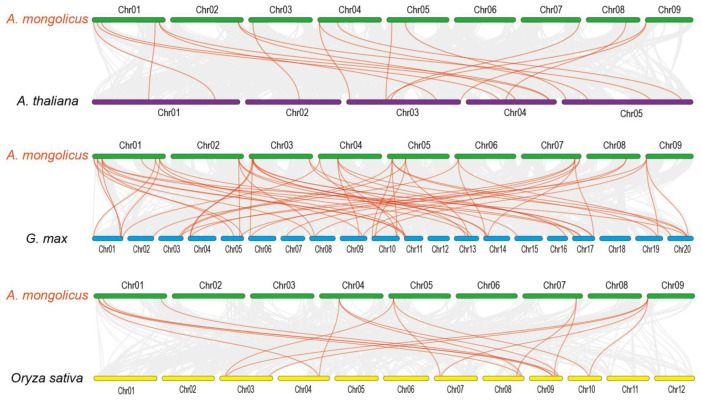
A synthetic analysis of the *A. mongolicus* genome in comparison to the genomes of one monocotyledon and two dicotyledon plants reveals alignment blocks represented by gray lines, while orange lines denote pairs of synthetic *HSF* genes.

The proportion of *HSF* genes common to *A. mongolicus* and other species may reflect the evolutionary relationships within the *AmHSF* genes. We examined the collinearity between the *HSF* genes of *A. mongolicus* and three representative species: one monocot, *O. sativa*, and two dicots, *A. thaliana* and *G. max* (Figure 6). A total of ninety direct homologous pairs (including one *A. mongolicus* gene that corresponds to multiple *G. max* genes) were obtained. Specifically, there were 19, 55, and 16 pairs of direct homologous genes in *A. thaliana*, *G. max*, and *O. sativa*, respectively. *G. max* and *A. mongolicus* exhibit the highest number of direct homologous genes, likely due to both species being part of the Leguminosae family. The aforementioned findings indicate that these genes likely have a significant role in the evolution of gene families. Throughout the evolutionary process, the majority of HSF genes in *A. mongolicus* exhibited more than two direct homologs in *G. max*, further implying that *G. max* has undergone a greater number of whole gene duplication events.

### 3.6. Analysis of Cis-Elements on Promoter of the HSF Gene in A. mongolicus

**Figure 7:**
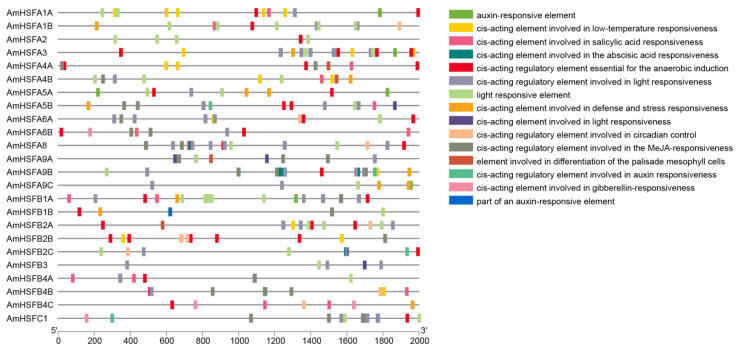
The schematic representation of *Cis*-acting elements within the 2000 bp promoter region upstream of the start codon of *AmHSF* genes illustrates distinct *Cis*-acting elements, each denoted by uniquely colored boxes.

*Cis*-regulatory elements play a crucial role in gene expression regulation. To gain further insights into the distribution of *Cis*-regulatory elements in the promoter of *AmHSF*, 2000 bp upstream of the ATG were *Cis*-regulatory elements in the promoters of *AmHSF* genes, encompassing com extracted from the genome file of *A. mongolicus.* The results revealed a plethora of hormone- and stress-relateponents involved in various responses, such as abscisic acid, MeJA, salicylic acid, gibberellin, auxin, light, low temperature, defense, stress, and anaerobic induction, and we identified elements associated with circadian rhythm control and differentiation of mesophyll cells (Figure 7). Additionally, we observed that among the genes *AmHSFA2*, *AmHSFA5A*, *AmHSFA9C*, *AmHSFB3*, and *AmHSFB4C*, there are fewer *Cis*-regulatory elements. Compared to other *AmHSF* genes, these genes exhibit a reduced number of cis-regulatory elements associated with plant hormones. Notably, the *AmHSFA2* gene did not contain any *Cis*-regulatory elements related to plant hormones.

### 3.7. Expression Profiling of the A. mongolicus HSF Gene under High-Temperature Stress

**Figure 8:**
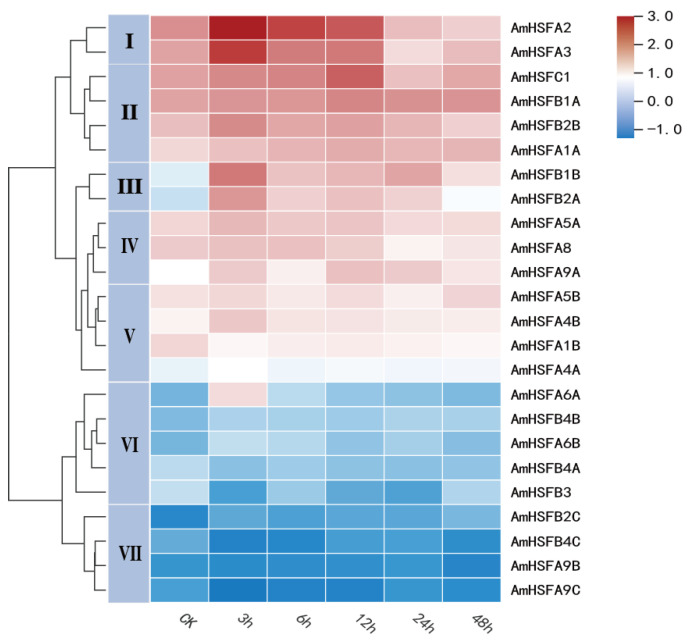
Heat maps of 24 *AmHSF* genes in different periods under heat stress. Red means high expression level; blue means low expression level. The FPKM value of each *AmHSF* was normalized by the log_10_ algorithm.

According to the transcriptome study results, we identified a total of 24 *AmHSFs* and analyzed their expression patterns under heat stress (Figure 8). The analysis results revealed diverse expression patterns among the 24 *AmHSFs* under different durations of 45 °C. Nearly half of the *AmHSFs* exhibited the highest relative expression levels at 3 h, such as *AmHSFA2*, *AmHSFA3*, *AmHSFA4A*, *AmHSFA4B*, *AmHSFA5A*, *AmHSFA6A*, *AmHSFA6B*, *AmHSFB1B*, *AmHSFB2A*, *AmHSFB2B*, and *AmHSFB4B*. Most *AmHSF* genes showed low expression level in the control group. Moreover, within the same subgroup, certain genes exhibited analogous expression patterns under 45 °C, as exemplified by *AmHSFA9B* and *AmHSFA9C*. By analyzing the expression patterns of gene clusters, we categorized them into seven distinct groups. Genes in group 1 including *AmHSFA2* and *AmHSFA3* exhibited higher relative expression levels under different time treatments at 45 °C compared to other groups and reached the peak at 3 h of heat stress. The *AmHSFAs* (*AmHSFA1A*, *AmHSFB1A*, *AmHSFB2B*, and *AmHSFC1*) of group 2 showed a higher level with the increase of heat stress. The *AmHSFAs* (*AmHSFB1B* and *AmHSFB2A*) of group 3 were very low without heat stress and reached the peak after 3 h of stress. The expression levels of genes in group 4 (*AmHSFA5A*, *AmHSFA8*, and *AmHSFA9A*) and group 5 (*AmHSFA1B*, *AmHSFA4A*, *AmHSFA4B*, and *AmHSFA5B*) increased under different time treatments at 45 °C, but the increase was not obvious. The expression levels of genes in group 6 and group 7 were very low under heat stress. Especially after heat stress, the expression levels of genes in group 7 (*AmHSFA9B*, *AmHSFA9C*, *AmHSFB2C*, and *AmHSFB4C*) showed a decreasing trend compared with the control group. It is noteworthy that regardless of the changes in relative expression levels of *AmHSF* genes under different durations of treatment at 45 °C, the majority of these genes exhibited a trend toward converging relative expression levels with the control group at 48 h of high-temperature stress.

### 3.8. GO Annotation and Enrichment Analysis of A. mongolicus HSF Genes

**Figure 9:**
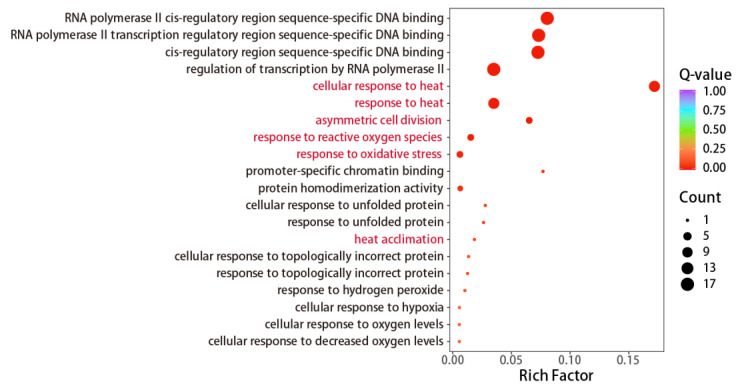
Bubble map of GO enrichment analysis of *AmHSF* genes: The horizontal axis represents enrichment factors, the size of the circle represents the number of genes for a given GO annotation, the red font represents the main functional channels involved.

To gain further insights into the biological functions of these *AmHSFs* in *A. mongolicus*, we selected 24 *AmHSFs* from the transcriptome database and performed GO annotation and enrichment analysis (Figure 9). In comparison to the comprehensive GO database, *AmHSFs* were significantly enriched in 18 biological processes, 1 cellular component, and 6 molecular functions. The result showed that *AmHSFs* were mainly involved in biological processes including the regulation of cellular response to heat, response to heat, heat acclimation, asymmetric cell division, response to reactive oxygen species, and response to oxidative stress. The GO analysis results indicate the crucial involvement of HSF genes in responding to different stages of high-temperature stress.

### 3.9. qRT-PCR Analysis of AmHSFs under High-Temperature Stress

**Figure 10:**
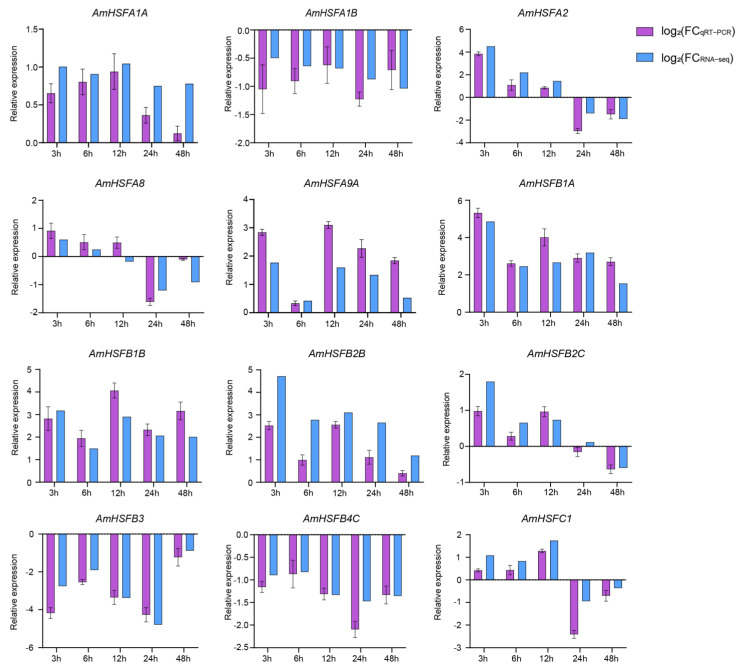
Correlation between qRT-PCR and RNA-seq expression based on 12 randomly selected *AmHSF* genes: the purple column represents expression abundance in qRT-PCR of selected AmHSFs; the blue column represents expression abundance in RNA-seq of selected *AmHSFs*.

To validate the RNA-seq results, the qRT-PCR method was utilized to examine the expression patterns of 12 *AmHSFs* that were chosen from 24 *AmHSFs*, including *AmHSFA1A*, *AmHSFA1B*, *AmHSFA2*, *AmHSFA8*, *AmHSFA9A*, *AmHSFB1A*, *AmHSFB1B*, *AmHSFB2B*, *AmHSFB2C*, *AmHSFB3*, *AmHSFB4C*, and *AmHSFC1*, under 45 °C high-temperature stress. Additionally, we conducted a correlation analysis between the qRT-PCR data normalized using the log_2_(qRT-PCR) algorithm and the RNA-seq data normalized using the log_2_(FoldChange) algorithm (Figure 10). The results indicated a strong correlation between qRT-PCR and RNA-seq data for all selected *AmHSFs* under the same stress conditions, demonstrating their similar expression trends.

## 4. Discussion

The presence of HSF transcription factors is extensive throughout plant species, playing a crucial role in plant growth, development, and responses to adversity. This study identified HSF gene families for the first time after the release of complete *A. mongolicus* genome data, leading to the discovery of 24 *HSF* genes. While the abundance of HSF genes varies widely across organisms, plants have more HSFs than animals and other microorganisms [33]. The 24 *AmHSFs* reported in this study are comparable in number to those reported in other leguminous members; for instance, common bean has 30 *HSFs* [34], alfalfa has 16 *HSFs* [13], and mung bean has 24 *HSFs* [35].

The plant HSF DBD (DNA-binding domain) is highly conserved and primarily located at the N-terminus, facilitating precise targeting and recognition of the promoter regions of heat stress elements (HSEs) [16,36,37]. Through multiple sequence alignments and compositional analyses of structural domains, it has been established that the DBD domain, consisting of three coiling bundles (α1, α2, and α3) and four anti-parallel ß-sheets, is present in all AmHSF proteins (Figure 2A). The oligomerization domains of different proteins exhibit characteristic features, with the HR-A/B regions in HSF proteins of the A, B, and C classes containing 21, 0, and 7 amino acid residues, respectively (Figure 2B). Interestingly, some AmHSF proteins also harbor additional conserved structural domains. Whether these additional domains serve as markers for the functional divergence of the gene family requires further experimental validation.

Phylogenetic tree analysis suggested that all major subclasses of HSFs in *Arabidopsis* can be found in *A. mongolicus*, except the A7 subclass (Figure 1). This may be due to differences between species. Based on homologous matching and multi-alignment, the *AmHSF* genes can be further classified into three groups: A, B, and C. Their clustering pattern resembles the *AtHSF* genes of *A. thaliana*, with the A group containing the highest number of genes and the C group having the least [19]. This suggests that although HSF proteins have evolved independently in different species, they still share common characteristics. Most *A. mongolicus* HSF proteins cluster with *G. max* HSF proteins rather than *A. thaliana* HSF proteins, indicating a strong evolutionary connection between *A. mongolicus* and *G. max*. We inferred that this is caused by the fact that both species belong to Leguminosae.

Apart from AmHSFB3, which is localized in the cytoplasm, all other HSF proteins are predominantly found in the cell nucleus, indicating distinct physicochemical properties between AmHSFB3 and other AmHSF proteins. Characteristics of genes, including distribution of exons and introns, gene length, and GC content, are significant factors influencing evolutionary events such as whole-genome duplication (WGD) [19]. Analysis of gene structure indicates that *A. mongolicus HSF* genes possess at least one intron, a characteristic also seen in the *HSF* gene families of other leguminous plants, including soybean, green bean, and mung bean. However, it has been observed that BoHsf34 lacks introns in *Brassica oleracea* [38]. Consequently, a hypothesis has been proposed, suggesting that the presence of genes with at least one intron is a distinctive trait of the HSF gene family in leguminous plants.

Protein motif analysis identified some functionally unknown and uniquely distributed motifs in *A. mongolicus* HSF proteins, suggesting a potential association with the functional diversity of HSF proteins. Across various plant species, there are variations in the composition, arrangement, and abundance of conserved motifs [27]. Analysis of conserved motifs has revealed that members of the same clade often share similar sequence structures. For instance, Motif 5 was found exclusively in Class A and Class C proteins; Motif 6 was present only in Class B proteins; Motif 7 appeared solely in Class A proteins; Motif 8 was identified in the A1, A4, and A5 subgroups; Motif 9 was primarily distributed in the A6 and A9 subgroups; and Motif 10 was unique to the A9 subgroup proteins. This structural specificity might contribute to the functional distinctiveness among different clades.

Gene duplication events significantly influence the formation of gene families by providing fundamental substrates for the emergence of novel functional genes [39]. Gene duplication primarily occurs via tandem and fragmental duplication mechanisms [40]. Within the *A. mongolicus* genome, the 24 *AmHSF* genes comprise 2 homologous gene pairs, all of which have experienced whole-genome duplication (WGD) or fragmental duplication events and have been subjected to significant purifying selection pressure. These observations suggest that WGD or fragmental duplication plays a crucial role in the amplification of the *AmHSF* gene family in *A. mongolicus* (Figure 5).

*Cis*-regulatory elements refer to nucleotide sequences positioned either upstream or downstream of genes, which regulate transcriptional levels of associated genes [41]. In plants, these elements play a critical role by binding to specific transcription factors during responses to diverse developmental processes and stress conditions [42]. In this study, certain *AmHSFs* encompass drought-responsive elements (MBS), anaerobic induction response elements (ARE), and low-temperature response elements (LTR), indicating the potential involvement of this gene family in various abiotic stimuli [13]. As reported in mung beans, all genes possess multiple drought-response elements (DREs) and ABA-responsive elements (ABREs) [35]. The *PvHSFA4A* gene in alfalfa contains the DRE1 dehydration response element [43]. In this study, we found the presence of the DRE1 dehydration response element in the *AmHSFA3* and *AmHSFA5B* genes. This suggests that the *AmHSFA3* gene and the *AmHSFA5B* gene may be the key transcription factors responsible for the dehydration response (Table 2).

In the gene expression profile generated from FPKM values of transcriptomic data, we observed that several genes (including *AmHSFB3*, *AmHSFB2C*, *AmHSFB4A*, *AmHSFB4C*, *AmHSFBA9B*, and *AmHSFA9C*) exhibited downregulated expression following high-temperature stress treatment. This downregulation may be attributed to the suppressive role of these *AmHSF* genes, which are subsequently activated through downstream genes in response to heat stress (Figure 8).

In the GO annotation and enrichment analysis, we observed that the heat acclimation pathway exhibited a low Q-value and count (with only one gene enriched). This may be attributed to the relatively short duration of exposure (48 h) to 45 °C heat stress of *A. mongolicus*. During this time, the relevant genes may have begun to respond but had not yet achieved full acclimation. To enrich additional genes within the heat acclimation pathway, a longer duration of exposure to 45 °C heat stress may be required for *A. mongolicus* (Figure 9).

In the correlation analysis presented in Figure 10, we compared qRT-PCR data normalized using the log_2_(qRT-PCR) algorithm with RNA-seq data normalized using the log_2_(FoldChange) algorithm. We found that the expression levels of certain genes did not perfectly align. This discrepancy may be due to potential errors inherent in both RNA-seq and qRT-PCR methodologies. Nonetheless, the overall trend demonstrates a high degree of consistency, thereby supporting the reliability of the RNA-seq data.

## 5. Conclusions

From the genomic information of *A. mongolicus*, a total of 24 *AmHSFs* were identified and classified into 3 groups and 13 subgroups. The physicochemical properties analysis demonstrated significant variations of physicochemical characteristics in these 24 AmHSF proteins. Their DNA-binding and oligomerization domains exhibit similar features to other species. *Cis*-acting elements and GO enrichment analyses indicated the potential involvement of *AmHSF* genes in diverse transcriptional regulatory mechanisms associated with stress responses during plant growth. Furthermore, expression profile analysis revealed significant differential expression of *AmHSF* genes at different time points under high-temperature stress, highlighting their crucial roles in responding to heat stress. In summary, the bioinformatics analysis and expression studies conducted on *AmHSF* genes enhance our comprehension of the critical role played by HSFs in the heat stress response of *A. mongolicus*. This research providing a valuable foundation for exploring and utilizing *AmHSF* to improve the heat stress tolerance of other crops.

## Figures and Tables

**Table 1:** Prediction of physicochemical properties of AmHSF.

Name	Gene ID	Size (aa)	MW (kDa)	PI	Stability	AI	GRAVY	Predicted Location
AmHSFA1A	Chr01.g02523.m1	512	56,088.54	4.86	U	68.79	−0.582	Nuclear
AmHSFA1B	Chr04.g17440.m1	489	54,257.51	5.29	U	68.55	−0.585	Nuclear
AmHSFA2	Chr03.g12796.m1	403	45,411.64	5.12	U	72.78	−0.648	Nuclear
AmHSFA3	Chr05.g25548.m1	517	58,431.1	4.87	U	68.8	−0.635	Nuclear
AmHSFA4A	Chr02.g11467.m1	398	45,240.57	5.33	U	70.75	−0.716	Nuclear
AmHSFA4B	Chr08.g41187.m1	398	45,509.92	5.5	U	71.76	−0.682	Nuclear
AmHSFA5A	Chr08.g41064.m1	487	54,911.89	5.86	U	64.31	−0.762	Nuclear
AmHSFA5B	Chr01.g04780.m1	169	18,980.93	6.19	S	55.38	−0.705	Nuclear
AmHSFA6A	Chr09.g43173.m1	383	44,040.33	5.41	U	59.84	−0.884	Nuclear
AmHSFA6B	Chr05.g23740.m1	361	41,696.03	5.08	U	75.54	−0.722	Nuclear
AmHSFA8	Chr01.g00197.m1	410	46,486.16	4.86	U	71.05	−0.68	Nuclear
AmHSFA9A	Chr08.g42499.m1	436	49,066.2	5.3	U	72.13	−0.604	Nuclear
AmHSFA9B	Chr03.g16772.m1	353	40,735.42	5.94	U	82.49	−0.642	Nuclear
AmHSFA9C	Chr03.g16773.m1	381	43,963.71	5.23	U	84.07	−0.631	Nuclear
AmHSFB1A	Chr01.g05312.m1	561	63,113.67	6	S	62.23	−0.816	Nuclear
AmHSFB1B	Chr01.g01208.m1	266	29,358.79	8.18	S	64.51	−0.694	Nuclear
AmHSFB2A	Chr01.g00693.m1	291	32,464.26	5.03	U	71.68	−0.66	Nuclear
AmHSFB2B	Chr04.g19853.m1	360	39,629.05	6.09	U	65	−0.669	Nuclear
AmHSFB2C	Chr01.g03279.m1	202	23,495.74	7.7	U	67.57	−0.708	Nuclear
AmHSFB3	Chr09.g43837.m1	132	15,105.3	6.4	U	92.2	−0.204	Cytoplasm
AmHSFB4A	Chr03.g12658.m1	370	41,648.89	8.87	U	71.11	−0.641	Nuclear
AmHSFB4B	Chr07.g37622.m1	378	42,610.86	7.26	U	68.33	−0.556	Nuclear
AmHSFB4C	Chr06.g29087.m1	270	31,458.64	6.83	U	70.41	−0.724	Nuclear
AmHSFC1	Chr07.g38211.m1	348	39,295.4	5.81	U	67.84	−0.556	Nuclear

**Table 2:** *Cis*-elements present in the promoters of *AmHSF* genes.

Gene	ABRE	ARE	DRE1	ERE	LTR	MBS	MRE	Myb	MYC	P-Box	STRE
*AmHSFA1A*	1	2	0	3	5	0	0	3	2	0	1
*AmHSFA1B*	4	1	0	1	0	0	1	7	7	0	5
*AmHSFA2*	0	1	0	3	0	1	1	7	6	0	1
*AmHSFA3*	11	4	1	0	3	0	0	10	3	0	2
*AmHSFA4A*	0	3	0	1	2	0	1	4	2	0	0
*AmHSFA4B*	1	0	0	2	2	0	0	6	2	0	2
*AmHSFA5A*	0	2	0	1	0	0	1	7	3	0	4
*AmHSFA5B*	3	2	1	2	0	0	0	10	2	0	2
*AmHSFA6A*	5	2	0	1	1	0	0	8	4	0	3
*AmHSFA6B*	0	2	0	0	0	0	0	2	4	2	2
*AmHSFA8*	7	2	0	1	0	0	1	4	4	1	3
*AmHSFA9A*	1	0	0	1	0	0	0	5	4	0	2
*AmHSFA9B*	8	1	0	2	1	1	3	5	4	0	2
*AmHSFA9C*	2	0	0	5	0	0	1	5	5	0	0
*AmHSFB1A*	3	2	0	0	0	1	0	9	4	1	2
*AmHSFB1B*	1	1	0	2	0	0	0	1	2	0	1
*AmHSFB2A*	4	4	0	1	1	1	1	8	5	0	2
*AmHSFB2B*	0	5	0	0	2	2	1	7	5	0	3
*AmHSFB2C*	3	1	0	1	0	0	0	2	6	2	0
*AmHSFB3*	1	0	0	2	0	0	0	3	4	1	2
*AmHSFB4A*	2	1	0	0	0	0	2	4	4	0	1
*AmHSFB4B*	2	1	0	2	1	0	1	1	2	0	1
*AmHSFB4C*	0	1	0	1	0	0	0	4	6	0	4
*AmHSFC1*	4	1	0	4	0	0	0	2	4	0	3

The abbreviated letters in the header represent different acting elements. ABRE: ACGTG, ARE: AAACCA, DRE1: ACCGAGA, ERE: ATTTTAAA, LTR: CCGAAA, MBS: CAACTG, MRE: AACCTAA, Myb: TAACTG, MYC: CATGTG, P-box: CCTTTTG, STRE: AGGGG.

## Data Availability

The sequences of identified HSFs and Supplemental Tables are included in this published article and its Supplementary Information Files. The sequencing data have been deposited in BIG Sub (https://ngdc.cncb.ac.cn/gsub/submit/bioproject/list, accessed on 5 April 2024), with the accession number of PRJCA022968.

## Supplementary Materials

The following supporting information can be downloaded at: https://www.mdpi.com/article/10.3390/cimb46100678/s1, Table S1: IDs of AtHSF and GmHSF proteins; Table S2: the primer sequences of qPCR.

## References

[1] Zhu J.K. (2016). Abiotic Stress Signaling and Responses in Plants. Cell.

[2] Wang L., Liu Y., Chai M., Chen H., Aslam M., Niu X., Qin Y., Cai H. (2021). Genome-wide identification, classification, and expression analysis of the HSF gene family in pineapple (*Ananas comosus*). PeerJ.

[3] Chung E., Kim K.M., Lee J.H. (2013). Genome-Wide Analysis and Molecular Characterization of Heat Shock Transcription Factor Family in Glycine max-ScienceDirect. J. Genet. Genom..

[4] Ali S., Rizwan M., Arif M.S., Ahmad R., Hussain A. (2020). Approaches in Enhancing Thermotolerance in Plants: An Updated Review. J. Plant Growth Regul..

[5] Li X.T., Feng X.Y., Zeng Z., Liu Y., Shao Z.Q. (2021). Comparative Analysis of HSF Genes From Secale cereale and its Triticeae Relatives Reveal Ancient and Recent Gene Expansions. Front. Genet..

[6] Wiederrecht G., Seto D., Parker C.S. (1988). Isolation of the gene encoding the S. cerevisiae heat shock transcription factor. Cell.

[7] Scharf K.D., Rose S., Zott W., Schöffl F., Schöff F. (1990). Three tomato genes code for heat stress transcription factors with a region of remarkable homology to the DNA-binding domain of the yeast HSF. EMBO J..

[8] Hübel A., Schffl F. (1994). Arabidopsis heat shock factor: Isolation and characterization of the gene and the recombinant protein. Plant Mol. Biol..

[9] Yamanouchi U., Yano M., Lin H., Ashikari M., Yamada K. (2002). A rice spotted leaf gene, SpI7, encodes a heat stress transcription factor protein. Proc. Natl. Acad. Sci. USA.

[10] Guo J., Wu J., Ji Q., Wang C., Luo L., Yuan Y., Wang Y., Wang J. (2008). Genome-wide analysis of heat shock transcription factor families in rice and Arabidopsis. J. Genet. Genom..

[11] Wang C., Zhang Q., Shou H.X. (2009). Identification and expression analysis of OsHsfs in rice. J. Zhejiang Univ..

[12] Lin Y.X., Jiang H.Y., Chu Z.X., Tang X.L., Zhu S.W., Cheng B.J. (2011). Genome-wide identification, classification and analysis of heat shock transcription factor family in maize. BMC Genom..

[13] Wang F., Dong Q., Jiang H., Zhu S., Chen B., Xiang Y. (2012). Genome-wide analysis of the heat shock transcription factors in *Populus trichocarpa* and *Medicago truncatula*. Mol. Biol. Rep..

[14] Zhou S., Zhang P., Jing Z., Shi J. (2013). Genome-wide identification and analysis of heat shock transcription factor family in cucumber (*Cucumis sativus* L.). Plant Omics.

[15] Xue G.-P., Sadat S., Drenth J., McIntyre C.L. (2014). The heat shock factor family from Triticum aestivum in response to heat and other major abiotic stresses and their role in regulation of heat shock protein genes. J. Exp. Bot..

[16] Ma J., Zhang G., Ye Y., Shang L., Hong S., Ma Q., Zhao Y., Gu C. (2022). Genome-Wide Identification and Expression Analysis of HSF Transcription Factors in Alfalfa (*Medicago sativa*) under Abiotic Stress. Plants.

[17] Fragkostefanakis S., Mesihovic A., Simm S., Paupière M.J., Hu Y., Paul P., Mishra S.K., Tschiersch B., Theres K., Bovy A. (2016). HsfA2 controls the activity of developmentally and stress-regulated heat stress protection mechanisms in tomato male reproductive tissues. Plant Physiol..

[18] Lili Z., Wei C., Jian W., Jingjin Y., Zhimin Y., Bingru H. (2018). Characterization and Functional Analysis of FaHsfC1b from *Festuca arundinacea* Conferring Heat Tolerance in Arabidopsis. Int. J. Mol. Sci..

[19] Guo M., Liu J.-H., Ma X., Luo D.-X., Gong Z.-H., Lu M.-H. (2016). The plant heat stress transcription factors (HSFs): Structure, regulation, and function in response to abiotic stresses. Front. Plant Sci..

[20] Zhang N., Wang Y., Wang Z., Yue Z., Niu Y. (2021). Heat shock transcription factor family in plants: A review. Sheng Wu Gong Cheng Xue Bao.

[21] Shao K.-Z., Lyu X.-P., Li J.-L., Chen J., Zhao L.-Y., Ren W., Zhang J.-L. (2022). Biological characteristics of heat shock transcription factors and their roles in abiotic stress adaptation of higher plant. J. Appl. Ecol..

[22] Harrison C.J., Bohm A.A., Nelson H.C. (1994). Crystal structure of the DNA binding domain of the heat shock transcription factor. Science.

[23] Nover L., Scharf K.-D., Gagliardi D., Vergne P., Czarnecka-Verner E., Gurley W.B. (1996). The Hsf world: Classification and properties of plant heat stress transcription factors. Cell Stress Chaperones.

[24] Scharf K.-D., Berberich T., Ebersberger I., Nover L. (2012). The plant heat stress transcription factor (Hsf) family: Structure, function and evolution. Biochim. Biophys. Acta Gene Regul. Mech..

[25] Zhang M., Wang Z., Jian S. (2022). Functional Characterization of Heat Shock Factor (CrHsf) Families Provide Comprehensive Insight into the Adaptive Mechanisms of Canavalia rosea (Sw.) DC. to Tropical Coral Islands. Int. J. Mol. Sci..

[26] Döring P., Treuter E., Kistner C., Lyck R., Chen A., Nover L. (2000). The role of AHA motifs in the activator function of tomato heat stress transcription factors HsfA1 and HsfA2. Plant Cell.

[27] Wang X., Shi X., Chen S., Ma C., Xu S. (2018). Evolutionary origin, gradual accumulation and functional divergence of heat shock factor gene family with plant evolution. Front. Plant Sci..

[28] Jiao S., Yao W., Zhang N., Xu W. (2019). Research progress of heat stress transcription factors (HsFs) in horticultural plants. J. Fruit Sci..

[29] Dong X., Zhang J., Xin Z., Huang Y., Han C., Li Y., Lu Q. (2023). Ecological Stoichiometric Characteristics in Organs of *Ammopiptanthus mongolicus* in Different Habitats. Plants.

[30] Hua W., Guixia J., Qiong D. (2005). Research progress of abiotic stress tolerant mechanisms and application prospect of *Ammopiptanthus mongolicus* Maxim. Chin. Sci. Bull.

[31] Qu S., Jiao Y., Abraham L., Wang P. (2021). Correlation analysis of new soybean [*Glycine max* (L.) Merr] gene Gm15G117700 with oleic acid. Phyton.

[32] Chen C., Wu Y., Li J., Wang X., Zeng Z., Xu J., Liu Y., Feng J., Chen H., He Y. (2023). TBtools-II: A “one for all, all for one” bioinformatics platform for biological big-data mining. Mol. Plant.

[33] Andrási N., Pettkó-Szandtner A., Szabados L. (2021). Diversity of plant heat shock factors: Regulation, interactions, and functions. J. Exp. Bot..

[34] Zhang Q., Geng J., Du Y., Zhao Q., Zhang W., Fang Q., Yin Z., Li J., Yuan X., Fan Y. (2022). Heat shock transcription factor (Hsf) gene family in common bean (*Phaseolus vulgaris*): Genome-wide identification, phylogeny, evolutionary expansion and expression analyses at the sprout stage under abiotic stress. BMC Plant Biol..

[35] Li S., Wang R., Jin H. (2019). Molecular characterization and expression profile analysis of heat shock transcription factors in mungbean. Front. Genet.

[36] Zhang H., Yang J., Chen Y., Mao X., Wang Z., Li C. (2013). Identification and expression analysis of the heat shock transcription factor (HSF) gene family in *Populus trichocarpa*. Plant Omics.

[37] Li W., Wan X.-L., Yu J.-Y., Wang K.-L., Zhang J. (2019). Genome-wide identification, classification, and expression analysis of the Hsf gene family in carnation (*Dianthus caryophyllus*). Int. J. Mol. Sci.

[38] Lohani N., Golicz A.A., Singh M.B., Bhalla P.L. (2019). Genome-wide analysis of the Hsf gene family in Brassica oleracea and a comparative analysis of the Hsf gene family in *B. oleracea*, *B. rapa* and *B. napus*. Funct. Integr. Genom..

[39] Shen C., Yuan J. (2020). Genome-wide characterization and expression analysis of the heat shock transcription factor family in pumpkin (*Cucurbita moschata*). BMC Plant Biol..

[40] Agarwal P., Khurana P. (2019). Functional characterization of HSFs from wheat in response to heat and other abiotic stress conditions. Funct. Integr. Genom..

[41] Ducy P., Karsenty G. (1995). Two distinct osteoblast-specific cis-acting elements control expression of a mouse osteocalcin gene. Mol. Cell Biol..

[42] Wang R., Zhong Y., Liu X., Zhao C., Zhao J., Li M., Ul Hassan M., Yang B., Li D., Liu R. (2021). Cis-regulation of the amino acid transporter genes ZmAAP2 and ZmLHT1 by ZmPHR1 transcription factors in maize ear under phosphate limitation. J. Exp. Bot..

[43] Mallick B., Kumari M., Pradhan S., Acharya G., Naresh P., Das B., Shashankar P. (2022). Genome-wide analysis and characterization of heat shock transcription factors (Hsfs) in common bean (*Phaseolus vulgaris* L.). Funct. Integr. Genom..

