# Supplementary material for: Genome-Wide Identification and Expression Analysis of the HSF Gene Family in Ammopiptanthus mongolicus
Source: Curr Issues Mol Biol. 2024 Oct 14;46(10):11375–93. doi: 10.3390/cimb46100678 (PMC11505871; doi:10.3390/cimb46100678)
Supplement: Supplementary file 1 [file cimb-46-00678-s001.zip › cimb-3213768-supplementary.pdf]

Table S1 : IDs for AtHSF and GmHSF proteins are listed

GmHsF		AtHSF	
Glyma.01g185800	*GmHsf-01*	AT1G32330.1	*AtHSFA1D*
Glyma.01g015900	*GmHsf-02*	AT1G46264.1	*AtHSFB4*
Glyma.01g217400	*GmHsf-03*	AT1G67970.1	*AtHSFA8*
Glyma.01g233000	*GmHsf-04*	AT2G26150.1	*AtHSFA2*
Glyma.01g143500	*GmHsf-05*	AT2G41690.1	*AtHSFB3*
Glyma.03g135800	*GmHsf-06*	AT3G02990.1	*AtHSFA1E*
Glyma.03g191100	*GmHsf-07*	AT3G22830.1	*AtHSFA6B*
Glyma.04g052000	*GmHsf-08*	AT3G24520.1	*AtHSFC1*
Glyma.05g151800	*GmHsf-09*	AT3G51910.1	*AtHSFA7A*
Glyma.05g240500	*GmHsf-10*	AT3G63350.1	*AtHSFA7B*
Glyma.05g162300	*GmHsf-11*	AT4G11660.1	*AtHSFB2B*
Glyma.05g095900	*GmHsf-12*	AT4G13980.1	*AtHSFA5*
Glyma.08g119900	*GmHsf-13*	AT4G17750.1	*AtHSFA1A*
Glyma.08g047400	*GmHsf-14*	AT4G18880.1	*AtHSFA4A*
Glyma.08g108600	*GmHsf-15*	AT4G36990.1	*AtHSFB1*
Glyma.09g190600	*GmHsf-16*	AT5G03720.1	*AtHSFA3*
Glyma.09g206600	*GmHsf-17*	AT5G16820.1	*AtHSFA1B*
Glyma.09g143200	*GmHsf-18*	AT5G43840.1	*AtHSFA6A*
Glyma.10g237800	*GmHsf-19*	AT5G45710.1	*AtHSFA4C*
Glyma.10g066100	*GmHsf-20*	AT5G54070.1	*AtHSFA9*
Glyma.10g029600	*GmHsf-21*	AT5G62020.1	*AtHSFB2A*
Glyma.10g003100	*GmHsf-22*		
Glyma.10g244000	*GmHsf-23*		
Glyma.11g009800	*GmHsf-24*		
Glyma.11g056200	*GmHsf-25*		
Glyma.11g025700	*GmHsf-26*		
Glyma.13g180200	*GmHsf-27*		
Glyma.13g151200	*GmHsf-28*		
Glyma.13g225700	*GmHsf-29*		
Glyma.14g096800	*GmHsf-30*		
Glyma.15g086400	*GmHsf-31*		
Glyma.16g196200	*GmHsf-32*		
Glyma.16g091800	*GmHsf-33*		
Glyma.17g227600	*GmHsf-34*		
Glyma.17g174900	*GmHsf-35*		
Glyma.19g137800	*GmHsf-36*		
Glyma.20g150300	*GmHsf-37*		
Glyma.20g156800	*GmHsf-38*		

Table S2. The primer sequences of qPCR

Gene ID		primer sequences
AmeIF	Forward primer	CTGACATGCGCCGTAGGAACG
	Reverse primer	CCCTGCTTATGCCAGTCTTTT
AmHSFC1	Forward primer	AGCGAAACTGAAGGAGGAGC
	Reverse primer	CCCAAGCTGCTTCCTCTCTC
AmHSFA1B	Forward primer	GGAAAGTTTGGGCTGGAGGA
	Reverse primer	AGAAGCCCGGACTATGCATG
AmHSFB3	Forward primer	ATCCTGCCACTGACGATGTC
	Reverse primer	TATTCAGCTGGCGGACGAAG
AmHSFA1A	Forward primer	CAACAGCTTCGTGGTTTGGA
	Reverse primer	GTGATTGCTGTGCCTGTTGA
AmHSFA2A	Forward primer	TCTCCACCGCCATTTTACCT
	Reverse primer	CACAACCCTCACTTCCTTGC
AmHSFB1A	Forward primer	TGGAGGATGCTGACACCAAC
	Reverse primer	CAACGAGCTCCTTGTGTCCT
AmHSFA9B	Forward primer	AACTCTATGAAGCCTGGCGT
	Reverse primer	TGTTTCAACTCTGCACACCG
AmHSFA9A	Forward primer	CCTGGAGGCTGAAGTTGAGA
	Reverse primer	AGCCATGTCAGTCCCATCAA
AmHSFB2B	Forward primer	CACCGGGGCTGTTAGAAGAG
	Reverse primer	TCTTCTTGCGGCGTTACAGT
AmHSFB1B	Forward primer	AAACACAAGAGAGAGCGCCA
	Reverse primer	TCGCTGCTCTTCATCACAGG
AmHSFA8	Forward primer	GGCTGAACCGGGGAATATGT
	Reverse primer	ATTCCAGGCTCAGGTTGCTC
AmHSFB4C	Forward primer	ACTCCGATGACCAACCCAAT
	Reverse primer	GCCTCAGTCTTTCGTTGTCC
